# International myeloma working group immunotherapy committee recommendation on sequencing immunotherapy for treatment of multiple myeloma

**DOI:** 10.1038/s41375-024-02482-6

**Published:** 2025-01-27

**Authors:** Luciano J. Costa, Rahul Banerjee, Hira Mian, Katja Weisel, Susan Bal, Benjamin A. Derman, Maung M. Htut, Chandramouli Nagarajan, Cesar Rodriguez, Joshua Richter, Matthew J. Frigault, Jing C. Ye, Niels W. C. J. van de Donk, Peter M. Voorhees, Benjamin Puliafito, Nizar Bahlis, Rakesh Popat, Wee Joo Chng, P. Joy Ho, Gurbakhash Kaur, Prashant Kapoor, Juan Du, Fredrik Schjesvold, Jesus Berdeja, Hermann Einsele, Adam D. Cohen, Joseph Mikhael, Yelak Biru, S. Vincent Rajkumar, Yi Lin, Thomas G. Martin, Ajai Chari

**Affiliations:** 1 Division of Hematology and Oncology, Department of Medicine, University of Alabama at Birmingham, Birmingham, AL USA; 2 Division of Hematology and Oncology, University of Washington, Fred Hutchinson Cancer Center, Seattle, WA USA; 3 Department of Oncology, McMaster University, Hamilton, ON Canada; 4 University Medical Center Hamburg-Eppendorf, Hamburg, Germany; 5 Section of Hematology/Oncology, University of Chicago Medical Center, Chicago, IL USA; 6 Division of Myeloma, Department of Hematology & Hematopoietic Cell Transplantation, City of Hope Cancer Center, Duarte, CA USA; 7 Singapore General Hospital, Singapore, Singapore; 8 Division of Hematology and Medical Oncology, The Tisch Cancer Institute, Mount Sinai School of Medicine, New York, NY USA; 9 Department of Medicine, Division of Hematology & Oncology, Massachusetts General Hospital Cancer Center and Harvard Medical School, Boston, MA USA; 10 MD Anderson Cancer Center, University of Texas, Houston, TX USA; 11 Amsterdam UMC, Vrije Universiteit Amsterdam, Amsterdam, Netherlands; 12 Levine Cancer Institute, Atrium Health Wake Forest Baptist, Charlotte, NC USA; 13 Arnie Charbonneau Cancer Research Institute, University of Calgary, Calgary, Alberta Canada; 14 NIHR UCLH Clinical Research Facility, University College London Hospitals NHS Foundation Trust, London, UK; 15 National University Cancer Institute, Singapore, National University Health System; Cancer Science Institute of Singapore, National University of Singapore, Singapore, Singapore; 16 Royal Prince Alfred Hospital and University of Sydney, Sydney, NSW Australia; 17 Division of Hematology, Mayo Clinic, Rochester, MN USA; 18 Department of Hematology, Myeloma and Lymphoma Center, Shanghai Changzheng Hospital, Navy Medical University, Shanghai, China; 19 Oslo Myeloma Center, Department of Hematology, Oslo University Hospital, Oslo, Norway; 20 Greco-Hainsworth Centers for Cancer Research, Tennessee Oncology, Nashville, TN USA; 21 Department of Internal Medicine II, University Hospital, Wurzburg, Germany; 22 University of Pennsylvania, Philadelphia, Pennsylvania, PA USA; 23 Translational Genomics Research Institute, City of Hope Cancer Center, Phoenix, AZ USA; 24 International Myeloma Foundation, Studio City, CA USA; 25 UCSF Helen Diller Family Comprehensive Cancer Center, San Francisco, CA USA

**Keywords:** Cancer immunotherapy, Myeloma, Targeted therapies, Myeloma

## Abstract

T-cell redirecting therapy (TCRT), specifically chimeric antigen receptor T-cell therapy (CAR T-cells) and bispecific T-cell engagers (TCEs) represent a remarkable advance in the treatment of multiple myeloma (MM). There are several products available around the world and several more in development targeting primarily B-cell maturation antigen (BCMA) and G protein–coupled receptor class C group 5 member D (GRPC5D). The relatively rapid availability of multiple immunotherapies brings the necessity to understand how a certain agent may affect the safety and efficacy of a subsequent immunotherapy so MM physicians and patients can aim at optimal sequential use of these therapies. The International Myeloma Working Group conveyed panel of experts to review patient and disease-related factors affecting efficacy and safety of immunotherapy, summarize existing information on sequencing therapy and provide a series of core recommendations.

## Introduction

The management of multiple myeloma (MM) is being revolutionized by the introduction of T-cell redirecting therapies (TCRT), specifically chimeric antigen receptor T-cell (CAR T-cell) therapies [1–4] and bispecific T-cell engaging (TCE) antibodies [5–7]. The International Myeloma Working Group (IMWG) Immunotherapy Committee has previously published recommendations on the optimal use of these treatments in relapsed or refractory multiple myeloma (RRMM) [8, 9].

Most of the clinical trials that supported the regulatory approval of these immunotherapy agents included patients who had not previously been exposed to another TCRT. However, with an increasing number of TCRT targets and mechanisms of action coupled with increasing but differential levels of availability to these treatments across the world, more permutations of serial TCRTs will continue to emerge. It is thus necessary to better evaluate the impact of any given TCRT on tumor biology, the immune microenvironment, and provide guidance on optimal sequencing of these agents, considering the resultant impact on the efficacy and safety of the future treatments.

The IMWG Immunotherapy Committee conveyed a panel of 30 experts in March 2024 to review, summarize, and interpret existing evidence and provide practical management recommendations where appropriate. Experts formed teams to review data for specific subsections, anchored on the first immunotherapy and describing subsequent outcomes (for example BCMA-targeted TCE after a BCMA-targeted CAR T-cell therapy). We included evidence from prospective clinical trials and real-world (RW) series disseminated as peer-reviewed manuscripts or meeting abstracts. We organized the data according to first immunotherapy and summarized the outcomes of sequential therapy in dedicated tables. When necessary, we contacted authors for additional information. From the report of each team, we composed an initial document that was further developed through a series of teleconferences. The finalized document includes summary of the evidence and 9 consensus recommendations.

## Available immunotherapies

### Autologous CAR T-cells

For autologous CAR T-cell therapy, a patient undergoes apheresis of mononuclear cells at accredited centers that are then submitted to a manufacturing process which includes T-cell purification, CAR transfection (typically using a lentiviral vector), ex vivo expansion, and quality control. The CAR transgene encodes one or more antigen recognition domains derived from an antibody structure, a hinge region, a transmembrane domain and intracellular signaling domains. The CAR transgene integrates into the T-cell genome, thereby allowing for its long-term expression. CAR T-cells are infused into the patient following a short course of lymphodepleting chemotherapy (LDC), typically with fludarabine and cyclophosphamide. Once infused, CAR T-cells recognize and bind to the target antigen (e.g., B-cell maturation antigen, BCMA) which aids T-cell expansion and triggers activation leading ultimately to T-cell induced lysis of target cells.

The first available CAR T-cell therapy in MM was idecabtagene vicleucel (ide-cel) based initially on the results of the KarMMa trial [3], investigating the efficacy of ide-cel in patients with at least three prior lines of therapy including a proteasome inhibitor (PI), immunomodulatory agent (IMiD), and an anti-CD38 monoclonal antibody (mAb). The overall response rate (ORR) was 73%, including 33% who achieved complete response (CR) or better. The median progression-free survival (PFS) was 8.8 months, reaching 12.1 months for patients at the highest safe cell dose. The subsequent phase 3 KarMMa-3 study compared the efficacy of ide-cel versus certain standard-of-care (SOC) regimens for patients with two or more prior lines of therapy including a PI, IMiD, and anti-CD38 mAb [1]. Ide-cel was associated with superior PFS (HR 0.49, 95% CI 0.38–0.65) compared to SOC with a median PFS of 13.3 vs. 4.4 months, supporting the availability of ide-cel in earlier lines of therapy in some countries.

Ciltacabtagene autoleucel (cilta-cel) has two BCMA-binding domains per CAR aiming at enhanced activity. The CARTITUDE-1 was a phase 1b/2 trial investigating cilta-cel in MM patients who had received 3 or more prior regimens or had disease refractory to a PI and IMiD [4]. All CARTITUDE-1 participants were triple-class-exposed, meaning that they had received a PI, IMiD, and anti-CD38 mAb. The ORR was 98% with 83% of patients achieving stringent CR (sCR) and median PFS was 34.9 months [10]. While cilta-cel generated deep and durable responses, 6% of CARTITUDE-1 recipients subsequently developed Parkinsonism and 10% subsequently developed myeloid malignancies.

The subsequent phase 3 CARTITUDE-4 trial compared the efficacy of cilta-cel versus certain SOC regimens in patients with lenalidomide-refractory MM who had received 1-3 prior lines of therapy; of note, anti-CD38 mAb exposure was not required for this study [2]. At a median follow-up of 33.6 months, cilta-cel was associated with improved PFS (HR 0.29; 95% CI 0.22–0.39) compared to SOC. The median PFS was not yet reached with cilta-cel versus 11.8 months. Cilta-cel was also associated with improved overall survival (OS) compared to SOC (HR 0.55; 95% CI 0.39–0.79) [11]. Rates of Parkinsonism and secondary hematologic malignancies were 1% and 3% respectively. The results of CARTITUDE-4 have led to the approval of cilta-cel after 1 line of therapy for patients with lenalidomide-refractory MM in some countries.

### Bispecific T-cell engagers

TCEs, also known as bispecific antibodies, consist of synthetic antibodies with a F_ab_ domain with affinity for CD3 on T-cells coupled with one or more F_ab_ domains with affinity for the antigen of interest on the tumor cell surface. TCEs that contain an F_C_ portion often carry modifications to minimize complement activation and optimize pharmacokinetics [12]. Concomitant TCE binding to both targets facilitates trafficking of T-cells into the tumor microenvironment and is sufficient to trigger T-cell activation and lysis of the tumor cells resulting from the release of pro-apoptotic perforins and granzymes while activating other immune cells through pro-inflammatory cytokines [9].

Teclistamab, a BCMAxCD3 TCE, was first studied in the MajesTEC-1 trial in patients who had received at least 3 prior therapies and who were triple-class exposed [5]. Among 165 patients treated at the recommended phase 2 dose (RP2D), the ORR was 63% with 39% achieving ≥ CR, translating into a median PFS of 11.3 months [13]. Elranatamab, also a BCMAxCD3 TCE, was studied in a similar patient population in the MagnetisMM-3 trial with ORR of 61%, 35% ≥CR, and median PFS was 17.2 months [14].

Infections comprise the most important class of adverse events (AEs) with BCMAxCD3 TCEs. However, the risk of infections can be greatly reduced by anti-microbial prophylaxis and immunoglobulin replacement therapy (e.g., intravenous immunoglobulin) following existing consensus recommendations [15–17].

Talquetamab is currently the only approved non-BCMA TCRT, consisting of a GPRC5D (G protein–coupled receptor class C group 5 member D)xCD3 TCE. In the MonumenTAL-1 trial, talquetamab was used to treat triple-class-exposed patients with RRMM who had received at least 3 prior regimens. Among the 297 patients treated at the optimal doses and schedules, the ORR was 70–74% and 33–40% achieved ≥ CR. Responses to talquetamab can be durable with median PFS of 7.5–11.2 months across different RP2Ds [7, 18, 19]. While infectious complications were lower with talquetamab than with BCMAxCD3 TCEs, other unique on-target-off-tumor (OTOT) adverse events were seen such as dysgeusia, skin and nail changes in up to 63% of patients.

Teclistamab, elranatamab, and talquetamab, are all administered subcutaneously, and the risk and severity of cytokine release syndrome (CRS) and immune effector-cell associated neurotoxicity syndrome (ICANS) are mitigated using step-up dosing. Like nearly all prior studies in RRMM, initial TCE studies administered the RP2D at full intensity until progression of disease. However, given their unprecedented depth and durability of responses, the optimal duration and dose-intensity of TCEs is an area of active investigation. These agents are approved for RRMM with slightly different indications across several countries.

### Other and upcoming immunotherapies

Many emerging TCRTs use non-BCMA targets or dual antigen targeting. In a first-in-human study of MCARH109, an autologous GPRC5D-targeted CAR T-cell therapy, 17 patients with heavily pretreated MM were treated with a 71% response rate [20]. Another GPRC5D CAR T-cell produced 91% ORR in a population of 33 patients with RRMM, albeit in a setting where most had not previously been exposed to anti-CD38 mAb therapy [21]. A larger ongoing single-arm study of BMS-986393 (another GPRC5D-directed CAR T-cell product) treated 84 patients with RRMM and reported an ORR of 88% [22]. Of interest, OTOT toxicities such as dysgeusia appear to be less frequent, milder and of shorter duration with GPRC5D-directed CAR T-cell therapies than with GPRC5DxCD3 TCEs. Finally, dual-targeted autologous CAR T-cells – for example, targeting BCMA/GPRC5D or BCMA/CD19, are currently in development with promising preliminary results [23, 24].

Allogeneic “off-the-shelf” CAR T-cells are derived from healthy donors and aim to overcome two important limitations of autologous CAR T-cells, inadequate T-cell fitness and patient attrition that occurs due to long manufacturing intervals with autologous products. In a single-arm trial, ALLO-715 produced responses in 70% of patients treated at optimal doses but had a significant infection burden resulting from the use of enhanced LDC that included an anti-CD52 mAb [25]. Future allogeneic platforms will incorporate novel CAR manufacturing strategies (for example, non-viral DNA delivery technology or CRISPR editing) to negate the need for enhanced LDC. For instance P-BCMA-ALLO-1, which has shown responses even in patients previously exposed to autologous BCMA CAR T-cell, does not require CD52 blockade as part of lymphodepletion [26].

Cevostamab is an intravenous TCE that also targets FcRH5 (Fc receptor-homolog 5) [27]. In a phase 1 trial with 160 patients with RRMM, cevostamab at the highest dose level yielded a 55% ORR with main toxicities being CRS and neurotoxicity. Other TCEs in development include the trispecific antibodies JNJ-79635322 and ISB2001, which have BCMAxGPRC5DxCD3 and BCMAxCD38xCD3 binding to target two MM surface antigens simultaneously.

Finally, belantamab mafadotin (belamaf) is an anti-BCMA monoclonal antibody-drug conjugate (ADC) with monomethyl auristatin F (a microtubule-disrupting agent) as its payload. Belamaf elicits objective response in nearly one third of triple-class-exposed patients with RRMM [28]. Belamaf’s most pertinent toxicities are thrombocytopenia and keratopathy; the latter often leads to transient loss of visual acuity and is a cause of frequent dose reductions and omissions in real-world practice. Belamaf monotherapy failed to show superiority over pomalidomide and dexamethasone in the DREAMM-3 trial [29]. However, based on superior PFS with belamaf-based triplet regimens in the DREAMM-7 and DREAMM-8 trials compared to approved triplets [30, 31], it is expected that belamaf will become available in more countries in coming years. As such, while belamaf is not currently a consideration in the management of RRMM, existing real-world data around belamaf regarding TCRT sequencing were included in our analysis.

## Considerations on sequencing therapy

### Impact of conventional therapies on subsequent immunotherapy

Several MM therapies can potentially impede the effectiveness of subsequent CAR T-cells and TCEs. Most patients in key MM CAR T-cell trials were triple-class-exposed. Given the ubiquity of these agents in trial participants before CAR T-cell, these classes of drugs are known to be safe to use in patients who intend to subsequently pursue TCRT [1–4]. Of note, these trials typically required 2-week washout periods for all MM drugs prior to apheresis; however, the precise impact of the proximity of conventional agents to apheresis on manufacturing success and CAR T-cell efficacy is unknown. While preclinical data suggest that IMiDs may potentiate effector T-cell function [32–35], clinical validation of this hypothesis in the setting of TCRT is needed.

Bendamustine should be avoided prior to TCRT regardless of intervening time given evidence from several studies in lymphoma demonstrating a long-term detrimental impact on T-cells [36, 37].

### Holding and bridging therapy

Autologous CAR T-cell therapy was transformative in the management of RRMM. However, in its current iteration, the process often encounters barriers such as financial clearance, availability of a manufacturing slot, and apheresis capacity at the institution. Along with quality control, there are often multiple weeks between the time of apheresis to LDC and subsequent infusion of CAR T-cells (“vein to vein” time) and even more between the decision to pursue CAR T-cell therapy and infusion (“brain to vein” time) [3, 4, 38, 39]. This delay can jeopardize both the safety and feasibility of CAR T-cell infusion by allowing for uncontrolled tumor progression [40]. Indeed, one of the primary determinants of choosing between a CAR T-cell and a TCE for a given patient is the pace of disease, with those MM patients with fulminant disease progression being either unable to get to CAR T-cell infusion or having more complications during CAR T-cell treatment.

**Fig. 1:**
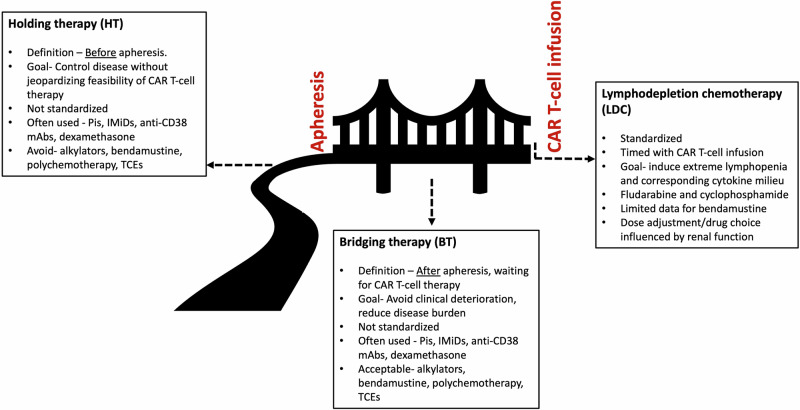
Distinction between holding therapy (HT), bridging therapy (BT), and lymphodepletion chemotherapy (LDC) in the care of the patient with multiple myeloma undergoing CAR T-cell therapy.

Bridging therapy (BT) is a plasma cell-directed therapy administered during the manufacturing period to provide disease control and allow for the safe administration of the CAR T-cells [39]. It is important to distinguish BT from MM treatment prior to apheresis (“holding therapy”, HT). BT is also different from LDC, a very standardized therapy, typically utilizing fludarabine in combination with cyclophosphamide, immediately prior to CAR T-cell infusion with the intention of inducing significant lymphopenia creating an optimal cytokine milieu that favors CAR T-cell expansion and persistence (Fig. 1) [41, 42].

The period between leukapheresis and CAR T-cell infusion can be remarkably tumultuous depending on the pace of disease progression. Patients with rapidly progressive disease may experience worsening cytopenia, progressive organ dysfunction and decline in functional status. Additionally, CAR T-cell infusion in the setting of high disease burden could be particularly associated with higher grade and severity of CRS and neurotoxicity [40], exemplified by the high rate of immune-mediated toxicities and even death in CARTITUDE-4 among patients who received cilta-cel as subsequent therapy upon progression prior to initiation of LDC [2].

In patients with heavily pretreated RRMM, if a decision to proceed with CAR T-cells is made at the time of disease progression on prior therapy, we recommend BT provided their disease has a high likelihood of morbidity within the following 4-6 weeks and there is a safe option with a reasonable expectation of response. Front-loaded regimens in which most drug exposure occurs within the first few weeks of the cycle are also preferred to allow for the resolution of toxicities prior to commencement of LDC.

The choice of systemic therapy must be individualized based on prior treatment history, exposure/refractoriness status, toxicity profile as well as comorbidities. In general, for patients with triple-class refractory disease, if carfilzomib-naïve, a carfilzomib-based combination is preferred if no clinical contraindication (either with low dose cyclophosphamide or pomalidomide) [43, 44]. Other options include low-dose weekly selinexor-based combination therapy, and off-label BCL2 inhibitor venetoclax among patients with t(11;14) [45–47]. For patients without recent alkylator exposure presenting with high disease burden and rapid disease progression, intensive multiagent alkylator combination is a consideration [48]. This approach has been shown to result in inferior outcomes compared to other therapies, highlighting the need for tailored treatment approach [49]. The recent availability of TCE provides an attractive novel strategy for BT. While using a BCMA directed TCE as BT prior to BCMA CAR T-cell may raise risk of decreased efficacy, using a GPRC5D-targeted TCE can bypass this risk by targeting a different antigen, with potential to eradicate disease clones which may be BCMA-low or -null, but still express GPRC5D [7]. Several groups have shown feasibility of this approach both as HT and BT, with excellent short term outcomes [50, 51]. If their use is being contemplated, we suggest beginning TCE immediately following apheresis. There is a paucity of data on duration of TCE usage as BT, and we suggest continuing BT until cells are successfully manufactured. While BT typically comprises systemic treatment, local palliative radiation therapy may be an option, particularly in patients with symptomatic extramedullary disease, or if there is risk of impending vital organ damage [52]. For patients who do not have triple-class refractory disease, or have anti-CD38 naïve or sensitive disease, an anti-CD38 mAb-based combination remains the preferred strategy given its safety and efficacy [2].

As the indications for CAR T-cells expand to earlier lines of treatment, there will be additional safe and efficacious systemic therapy options. Lastly, the development of rapid manufacturing and allogeneic platforms may ultimately obviate the need for BT [25, 53, 54].

### Host and tumor factors with implication for sequencing therapy

MM immunotherapies add an additional layer of complexity to sequencing considerations given that immune effector cells can be both quantitatively and qualitatively impaired by prior therapies. These changes are clinically relevant because T-cell characteristics are important determinants of response with both CAR T-cell and TCEs [55–58]. For example, pre-existing lymphopenia (which can occur when bendamustine or alkylators are used prior to apheresis) can worsen outcomes following CAR T-cell therapy or may preclude the successful manufacturing of an autologous product altogether [59]. In addition, continuous activation of T-cells with TCEs results in increased expression of inhibitory receptors possibly contributing to impaired antitumor efficacy in ex vivo experiments [56]. It remained however to be functionally demonstrated in clinical samples from TCE treated patients to what extent T cell exhaustion contributes to the reduced depth and duration of response to subsequent line of immunotherapy. As shown recently [60], variable factors including disease burden, targeted antigen density, mutation or shedding, as well as effector to target ratio (absolute T cell count to disease burden) and T cell dysfunction contribute to the reduce response seen in some patients sequentially treated with TCRT.

Independently of T-cell function, the sequential use of therapies targeting the same antigen can potentially predispose patients to inferior outcomes with the second therapy. For example, it is unlikely for BCMA-naïve patients to have inadequate BCMA expression on MM cells precluding response with BCMA-directed therapies. This is true even in patients with a monoallelic copy loss of *TNFRSF17* (on chromosome 16p, coding BCMA), which is present in 5-10% of BCMA-naïve patients [61]. In contrast, selective antigenic pressure with BCMA-targeted therapy has been linked to clinically significant changes (e.g., biallelic BCMA losses or monoallelic losses plus an extracellular mutation) in approximately 40% of patients [62, 63]. This risk also applies to belamaf, where biallelic loss of BCMA was recently described following treatment failure [64].

With GPRC5D, monoallelic copy loss on chromosome 12p is present in approximately 15% of patients prior to treatment [62, 63]. Perhaps owing to its non-essential role in plasma cell survival (unlike BCMA), convergent evolution with the emergence of several clones with either biallelic loss or monoallelic loss plus transmembrane or extracellular domain mutations appear to occur much more frequently under selective pressure of a GPRC5D-targeted TCE [62]. Similarly, GPRC5D antigen loss was described in most patients with progression after GPRC5D CAR T-cell therapy [20]. Importantly, mutations in BCMA or GPRC5D extracellular domains following the binding of a given therapy do not necessary preclude the binding of a different agent targeting that same antigen [62]. In the future, better diagnostic and predictive assays will help guide decision-making about the expected efficacy of sequential BCMA-targeted or GPRC5D-targeted therapies.

As such, recommendations about TCEs as HT or as BT prior to autologous CAR T-cell therapy must necessarily be made based on expert consensus and limited data. In general, TCEs should be avoided as HT if possible, regardless of target for two reasons. Firstly, TCEs (particularly in the initial 1-2 cycles) can lead to profound lymphopenia due to trafficking into the bone marrow and other sites of MM. Secondly, as detailed above, continuous TCE exposure can lead to T-cell dysfunction [56]. Patients proceeding to CAR T-cell therapy immediately after TCE have higher risk of manufacturing failure and less durable responses than comparable non-TCE-treated patients [65]. Given that treatment-free intervals can allow for recovery of T-cell number and function [66], a 4-week minimum washout period for TCE before apheresis is suggested regardless of TCE target. After apheresis, TCE therapy is reasonable as BT particularly if targeting a different antigen than CAR T-cell.

It should be noted that the biological impact of TCEs likely exceeds that of their half-lives. For example, the hypogammaglobulinemia associated with BCMA-targeted TCEs does not recover for even 6 months after discontinuation [67]. When the preceding TCE or ADC targets the same antigen as the prospective CAR T-cell product, RW data suggest an association between the interval since discontinuation of the prior agent and likelihood of success with CAR T-cell therapy [65, 68]. Such associations are of course confounded by disease characteristics, namely that patients with more aggressive MM will also have shorter treatment-free intervals due to underlying disease biology.

### Considerations on frailty and implications for sequencing therapy

Patient frailty is an important consideration in MM immunotherapy sequencing, perhaps even more so than with conventional MM therapies given the unique and multiorgan manifestations of immunotherapy-related toxicities such as CRS and neurotoxicity. Most frailty tools were developed and validated in the pre-immunotherapy era and therefore their prognostic ability as well as potential to predict the impact of toxicities unique to immunotherapy remain unknown. Given the principle of dynamic frailty in MM whereby effective therapies can improve patient fitness and abrogate the negative impact of frailty [69, 70], CAR T-cell therapy and TCE may potentially improve outcomes in frail patients if sequenced correctly. If sequenced incorrectly, on the other hand, patients can become too frail from disease-related manifestations or treatment-related toxicities to be considered for MM immunotherapies at all.

While frailty assessments are being increasingly incorporated into MM clinical trials [71], there remains a paucity of data regarding the impact of frailty on patients receiving TCRT. A post-hoc frailty analysis of the MagnetisMM-3 trial demonstrated similar ORRs (63% vs. 56%) with non-frail (*n* = 84) versus frail patients (*n* = 39) following elranatamab [72]. There were similar rates of CRS, neurotoxicity, and grade 3 or higher infections in the non-frail and frail groups, respectively. The impact of frailty on outcomes with T-cell engagers in the real-world is unknown; however, one analysis noted inferior overall response rates and PFS on multivariate analysis among those with poor performance status [73]. In a real-world series of patients receiving CAR T-cell therapy, there is a slightly higher rate of all grade ICANS noted among frail vs. non frail adults [74]. Similarly, registry data from the Center for International Blood and Marrow Research (CIBMTR) also noted that older age (>70 vs. <60) is associated with a higher rate of neurotoxicity [75]. Future larger series will be required to both verify and understand the potential mechanism of any toxicity seen among older and frailer adults treated with these agents.

## Specific sequencing scenarios

### TCRT after BCMA-targeted CAR T-cell therapy

Retreatment with the same CAR T-cell therapy does not appear to be an effective strategy (Table 1). In the KarMMa-1 study involving ide-cel, only 6 of 28 patients who were re-treated with ide-cel achieved a response [3]. Whether anti-drug antibodies play a role in response to retreatment is not clearly understood. In CARTITUDE-1, none of the 3 patients who were retreated with cilta-cel on study had a response [76]. Switching to a different BCMA-targeted CAR T-cell therapy may be an effective strategy based on RW data [65, 77]. For patients with an adequately long response to initial BCMA-targeted CAR T-cell therapy, it may be reasonable to attempt a different BCMA-targeted CAR T-cell therapy at relapse.

**Table 1:** T-cell redirecting therapy after BCMA-targeted CAR T-cell therapy.

	Study	*N*	ORR	CRR	PFS	DOR	Particularities/ unique toxicities
BCMA-targeted CAR T-cells							
Ide-cel	Munshi [3]	28	21%	0%	mPFS 1.0 mo.	n.a.	On trial, second administration of product
Cilta-cel	Martin [76]	3	0%	0%	n.a	n.a	On trial, second administration of product
Ide-cel	Ferreri [65]	5	80%	40%	n.r.	n.r.	RWE, 2 patients had received prior ide-cel
BCMA-targeted TCE							
Teclistamab	Touzeau [78]	15	53%	27%	mPFS 4.4 mo.	n.a	Dedicated MajesTEC-1 cohort
Elranatamab	Nooka [79]	36	53%	20%	mPFS 10 mo.	n.a	Pool from clinical trials
Teclistamab	Riedhammer [80]	21	33%	16%	mPFS 1.8 mo.	n.a	RWE, all prior CAR T-cells were ide-cel
Teclistamab	Dima [73]	43	63%	28%	n/a	n.a	RWE, 38/42 prior CAR T-cells were ide-cel
GPRC5D-targeted CAR T-cells							
MCARH109	Mailankody [20]	8	75%	n.a.	n.a	n.a	Cerebellar toxicity at the highest dose level
BMS-986393	Bal [22]	30	78%(a)	44%(a)	n.a.	n.a	Cerebellar toxicity at the highest dose level
OriCAR-017	Zhang [81]	5	100%	40%%	n.a.	n.a	Skin, nail, taste changes
n.a.	Xia [21]	9	100%	44%	n.a.	n.a	
GPRC5D-targeted TCE							
Talquetamab	Rasche [19]	56	71%	n.a.	n.a.	mDOR 12.3mo.	Subset of MonumenTAL-1 trial
Talquetmab plus daratumumab	Dholaria [82]	11	82%	n.a.	n.a.	n.a.	Subset of TRIMM-2 trial
Talquetamab, daratumumab and pomalidomide	Bhalis [83]	24	83%	71%	74% at 12 mo.	84% at 12 mo.	Subset of TRIMM-2 trial
Forimtamig	Harrison [84]	9	47%(b)	n.a.	n.a.	mDOR 9.0mo. (b)	
Other therapies							
Cevostamab	Kumar [85]	11	73%	27%	n.a	n.a.	Dedicated CAMMA2 trial cohort

(a) relates to the 39 patients with prior BCMA-targeted therapy, of which 30 received prior BCMA-targeted CAR T-cells. (b) For all 43 patients with prior BCMA-targeted therapy; *TCE* bispecific T-cell engager, *ORR* overall response rate, *CRR* complete response rate, *PFS* progression-free survival, *DOR* duration of response, *n.a.* not available, *n.r.* median not reached.

BCMA-targeted TCEs have been administered to patients following BCMA-targeted CAR T-cell therapies. In a dedicated cohort of the MajesTEC-1 trial, among 15 patients receiving teclistamab after prior BCMA-targeted CAR T-cell therapy the ORR was 53% yet median PFS was only 4.4 months [78]. As will be shown multiple times, ORR does not appear to be as adversely impacted as PFS in the setting of TCRT sequencing. For example, a pooled analysis of elranatamab in patients with previous BCMA CAR T-cell therapy demonstrated a 53% ORR but median PFS of 10.0 months [79]. RW data suggest a signal for better responses in patients with longer time since last BCMA-targeted therapy (> 6 months) [73, 80]. As a caveat, the usual confounding factors of more aggressive MM and impaired T-cell fitness still apply.

It has been surmised that changing the target of TCRT following relapse after BCMA-targeted CAR T-cell therapy may lead to better outcomes. GPRC5D has emerged as a leading target not only for TCEs but also for CAR T-cells. MCARH109, an investigational GPRC5D-targeted CAR T-cell, produced objective response in 6/8 patients with prior BCMA-targeted CAR T-cell therapy [20]. BMS-986393, also an investigational GPRC5D CAR T-cell, produced 78% ORR among 39 patients with prior BCMA-targeted therapy, of which 30 had prior CAR T-cells [22]. In summary, GPRC5D-targeted CAR T-cell therapy appears to be active in the majority of cases following BCMA-targeted therapies [20–22, 81].

There is some experience with the use of the GPRC5D-targeted TCE talquetamab after a BCMA-targeted CAR T-cell. Among 56 patients enrolled in MonumenTAL-1 with prior BCMA CAR T-cell therapy, 71% had an objective response and responses were durable (median duration of response 12.3 months) [19].

In the TRIMM 2 trial, talquetamab was combined with daratumumab for patients with RRMM leading to objective response in 9/11 patients with prior BCMA CAR T-cell therapy [82], while the combination of talquetamab, daratumumab and pomalidomide produced response in 20/24 patients [83]. Activity of GPRCD5D-targeted TCE on RRMM with prior BCMA-targeted CAR T-cell was also demonstrated in a phase 1 trial of forimtamig (Table 1) [84].

Cevostamab, an experimental FcRH5xCD3 TCE was tested in a CAMMA 2 cohort dedicated to patients with prior BCMA-targeted therapy. ORR was 73% among the 11 patients with prior BCMA-targeted CAR T-cell therapy [85].

In aggregate, these data seem to favor approaching a different therapeutic target upon progression after failure of a BCMA-directed CAR T-cell therapy. More data are needed to determine whether a particular MM cell antigen (e.g., GPRC5D or FcRH5) or a particular treatment modality (CAR T-cell versus TCE) is preferable in this setting. While BCMA-targeted TCEs may be reasonable to administer following relapse after BCMA CAR T-cell therapies, these are likely best suited for patients with longer interval since their previous CAR T-cell therapy [65, 68].

### TCRT after BCMA-targeted TCEs

There are minimal data on the use of TCRT after BCMA-targeted TCEs (Table 2). Cohort C of the CARTITUDE-2 study described 7 patients who received cilta-cel after previous BCMA TCE exposure; there were 4 responses, but median PFS was only 5.3 months [86]. There were also 3 deaths among this small group, including two from infectious causes. A multicenter RW study of ide-cel included 7 patients who had previously received a BCMA-targeted TCE; similarly, 6 patients achieved a response, but median PFS was only 2.8 months [65]. None of the reported clinical trials of BCMA-targeted TCE included patients with prior BCMA-targeted TCE, and this sequence has not yet been described in RW data.

**Table 2:** T-cell redirecting therapy after BCMA-targeted TCE.

	Study	*N*	ORR	CRR	PFS	DOR	Particularities/ unique toxicities
BCMA-targeted CAR T-cells							
Cilta-cel	Cohen [86]	7	57%	14%	mPFS 5.3mo.	mDOR 8.2 mo.	Dedicated CARTITUDE-2 cohort, 2 deaths dur to infection
Ide-cel	Ferreri [65]	7	86%	43%	mPFS 2.8 mo.	mDOR 2.8 mo.	RWE
BCMA-targeted TCE							
None reported							
GPRC5D-targeted CAR T-cells							
None reported							
GPRC5D-targeted TCE							
Talquetamab	Rasche [19]	26	58%	n.a.	mPFS 4.1mo.	n.a.	Subset of MonumenTAL-1 trial
Talquetmab plus daratumumab	Dholaria [82]	15	73%	n.a.	n.a.	n.a.	Subset of TRIMM-2 trial
Talquetamab, daratumumab, and pomalidomide	Bhalis [83]	29	83%	59%	69% at 12 mo.	70% at 12 mo.	Subset of TRIMM-2 trial, 23/29 with prior BCMA-targeted TCE
Other therapies							
None reported							

*TCE* bispecific T-cell engager, *ORR* overall response rate, *CRR* complete response rate, *PFS* progression-free survival, *DOR* duration of response, *n.a.* not available, *n.r.* median not reached.

A small number of patients with prior BCMA-targeted TCE were treated with the GPRC5D CAR T-cell BMS-986393, but results were not described for this subset. The phase 2 MonumenTAL-1 study of talquetamab reported on 26 patients who had previously received a BCMA-targeted TCE: ORR was 58% but median PFS only 4.1 months [19]. Of note, the ORR of talquetamab was lower in patients who had received a TCE as their immediate prior line of therapy compared to during any prior line of therapy (29% vs. 61%). In fact, RWE series have also reported ORR of only 33% when a TCE is followed by another TCE even with a different target [87]. This may be due to the presence of T-cell dysfunction rather than target antigen loss as the driver of relapse in these scenarios.

In general, data are scarce for immunotherapy following treatment with a BCMA-targeted TCE and the existing data seems to indicate compromised results with monotherapy – even when the subsequent therapy utilizes a different target. However, combination strategies may be more promising here. With the addition of daratumumab and pomalidomide to the GPRC5DxCD3 TCE talquetamab in a TRIMM-2 cohort, for example, responses were seen in 23 of 29 patients with prior TCE therapy (mostly BCMA-targeted) [83]. We await longer follow-up from future studies to assess the long-term success of this approach.

### TCRT after BCMA-targeted ADCs

As shown in Table 3, both the dedicated CARTITUDE-2 Cohort C trial of cilta-cel and RW ide-cel data demonstrate poor long-term outcomes with BCMA CAR T-cell in belamaf-exposed patients. In particular, while ORRs exceeded 60% in both cases, median PFS was only 9.5 months with cilta-cel and 3.2 months with ide-cel [65, 86].

**Table 3:** T-cell redirecting therapy after BCMA-targeted ADC.

	Study	*N*	ORR	CRR	PFS	DOR	Particularities/ unique toxicities
BCMA-targeted CAR T-cells							
Cilta-cel	Cohen [86]	13	62%	38%	mPFS 9.5mo	mDOR 11.5mo	Dedicated CARTITUDE-2 cohort
Ide-cel	Ferreri [65]	37	68%	22%	mPFS 3.2 mo.	mDOR 7.4 mo.	RWE
BCMA-targeted TCE							
Teclistamab	Touzeau [78]	29	55%	28%	mPFS 7.3 mo.	n.a.	Dedicated MajesTEC-1 cohort
Elranatamab	Nooka [79]	59	42%	19%	mPFS 3.9 mo.	n.a.	Pool from clinical trials
Teclistamab	Riedhammer [80]	23	74%	22%	n.a.	n.a.	RWE
Teclistamab	Dima [73]	23	61%	26%	n.a.	n.a.	
GPRC5D-targeted CAR T-cells							
None reported							
GPRC5D-targeted TCE							
Talquetamab	Jakubowiak [88]	8	75%	n.a.	n.a.	n.a.	Subset of MonumenTAL-1 trial
Forimtamig	Harrison [84]	28	47%(a)	n.a.	n.a.	mDOR 9.0mo. (a)	
Other therapies							
Cevostamab	Kumar [85]	10	60%	10%	n.a	n.a.	Dedicated CAMMA2 trial cohort

(a) For all 43 patients with prior BCMA-targeted therapy. *ORR* overall response rate, *TCE* bispecific T-cell engager, *CRR* complete response rate, *PFS* progression-free survival, *DOR* duration of response, *n.a.* not available, *n.r.* median not reached.

Among 29 patients with prior ADC therapy receiving teclistamab in the MajesTEC-1 trial, ORR was 55% with PFS of 7.3 months [78]. In RW data, the ORR to teclistamab ranges from 61–74% among patients with prior belamaf therapy [73, 80]. Finally, in a pooled analysis of 59 patients receiving elranatamab on trial, ORR was 42% but disease control was relatively short [79].

Very few patients with prior belamaf therapy were included in trials with GPRC5D-targeted CAR T-cells, and their outcomes have not been reported [20, 22]. In MonumenTAL-1, there were 6 responses among 8 patients with prior TCRT and prior belamaf therapy [88]. Patients with prior belamaf treatment were included in talquetamab-based combination trials, but their outcomes have not been reported separately [82, 89, 90]. In the CAMMA 2 study of cevostamab, there were similarly 6 responses among 10 patients with prior belamaf therapy [85]. In summary, while it appears clear that prior belamaf can compromise the durability of responses to subsequent BCMA-targeted TCRT, limited data to date do not suggest a similar impact on TCRTs using alternative targets.

### TCRT after GPRC5D-targeted therapies

Because the general development of BCMA-targeted therapies predates that of GPRC5D-targeted therapies, there is scarce information about subsequent TCRT after GPRC5D-targeted therapies (Table 4). An analysis of subsequent therapies for patients who received talquetamab as MonumenTAL-1 indicated responses in 7 of 9 patients receiving BCMA-targeted CAR T-cell therapy and 11 of 19 receiving BCMA-targeted TCE therapy [91]. Because antigen loss or modification appear to be common after GPRC5D-targeted therapy [20, 62], the use of a second GPRC5D-targeted agent is not recommended until additional data becomes available. The limited available information suggests that the efficacy of TCRT utilizing a different target is not strongly affected by prior GPRC5D-targeted TCRT [92].

**Table 4:** T-cell redirecting therapy after GPRC5D-targeted therapy.

	Study	N	ORR	CRR	PFS	DOR	Particularities/ unique toxicities
BCMA-targeted CAR T-cells							
Various	Sanchez [91]	9	78%	33%	n.a.	n.a.	Subsequent to talquetamab in MonumenTAL-1
Various	Joiner [92]	5	80%	n.a.	median 8.2 mo.	n.a.	Mixed of CAR T-cells and BCMA-targeted TCE
BCMA-targeted TCE							
Various	Sanchez [91]	19	58%	32%	n.a.	n.a.	Subsequent to talquetamab in MonumenTAL-1
GPRC5D-targeted CAR T-cells							
None reported							
GPRC5D-targeted TCE							
None reported							
Other therapies							
non-BCMA CAR T-cells (various)	Sanchez [91]	8	50%	38	n.a.	n.a.	Subsequent to talquetamab in MonumenTAL-1
non-BCMA TCE (various)	Sanchez [91]	4	75%	25	n.a.	n.a.	Subsequent to talquetamab in MonumenTAL-1

*TCE* bispecific T-cell engager, *ORR* overall response rate, *CRR* complete response rate, *PFS* progression-free survival, *DOR* duration of response, *n.a.* not available, *n.r.* median not reached.

### Non-TCRT after TCRT

Conventional therapies have not been systematically studied following TCRT failure. However, relapses after BCMA-targeted CAR T-cell therapy are sometimes salvageable with non-TCRT approaches. There is emerging pre-clinical data that XPO1 inhibitors have less detrimental and more potentiating effect on T-cells. In addition to direct cytotoxicity against malignant cells, XPO1 inhibitors may modulate the immune microenvironment to promote T-cell fitness and reduce markers of T-cell exhaustion. In the STOMP trial [93], a selinexor-based triplet or quadruplet combination induced responses in 7 of 11 patients (64%) with prior BCMA-targeted therapy. Another report indicated objective responses in 6 of 7 patients with relapse post BCMA-targeted CAR T-cells, treated with selinexor or selinexor combinations, including some durable responses [94].

CELMoDs (cereblon E3 ligase modulators) such as iberdomide and mezigdomide are oral investigational agents with higher potency than IMiDs. The CC-220-MM001 trial included a dedicated cohort with 41 patients previously exposed to BCMA-targeted therapy who received iberdomide plus dexamethasone [95]. The ORR was 34% and did not seem affected by the prior type of BCMA-targeted therapy; in responding patients, the median DOR was 7.5 months. Similarly, in 30 patients with prior BCMA-targeted therapy, mezigdomide plus dexamethasone produced an ORR of 50%, median DOR of 6.9 months, and median PFS of 5.4 months [96]. These all-oral doublet combinations warrant continued investigation and may be particularly helpful in scenarios where a different TCRT is not advisable or practical.

Since most of the patients with progression after TCRT currently have already received PIs, IMiDs and anti-CD38 mAbs, by employing combination regimens from these classes of drugs we expect ORR no higher than 30% and median PFS of approximately 4 months, consistent with prior observational studies [97, 98]. However, the use of cytotoxic chemotherapy +/− stem cell support, or salvage autologous stem cell transplantation, was associated with ORR of up to 50% in a single institution study [99] and remains an option for selected fit patients with cryopreserved stem cells available, especially in the setting of rapidly progressive disease or cytopenias and with the prospect of future access to clinical trials with agents with novel mechanism of action.

## Recommendations

Future evidence, both from RW data and ideally from randomized controlled trials, will be imperative to better guide decision-making around TCRT sequencing. Based on the evidence to date summarized above, this panel developed 9 recommendations for the optimal sequential use of immunotherapy in MM.

1. There are no concerns about proceeding with TCRT in patients receiving a PI, IMiD, (naked) monoclonal antibody, a corticosteroid or any combination of these classes as most recent line of therapy. When used as “holding therapy” before T-cell collection, we recommend a washout of 2 weeks between last dose of conventional agent and apheresis of mononuclear cells for CAR T-cell manufacturing. Similarly, we recommend a 2-week washout for these agents before the first dose of TCE.
2. If feasible, avoid collection of mononuclear cells for CAR T-cell manufacturing in patients receiving a TCE. If such sequence is the best option for the patient, aim for a minimum of 4-week washout between the last dose of the TCE and apheresis collection. Alternatively, if possible, consider T-cell collection prior to TCE initiation.
3. Avoid high-dose alkylators and bendamustine in patients for whom next therapy is likely to be CAR T-cell and/or a TCE.
4. Strongly consider bridging therapy after apheresis for CAR T-cell manufacturing in patients with high disease burden or at risk of developing morbidity from MM during the 4-6 weeks of manufacturing. The ideal bridging therapy will contain agent(s) without known resistance from the patient’s myeloma, be short, with low risk of infection or prolonged cytopenias.
5. Assuming equal access, in patients who are reasonable candidates to both BCMA-targeted ADC and BCMA-targeted TCRT, we recommend pursuing TCRT first given its higher activity and lower efficacy of TCRT after prior BCMA-targeted ADC.
6. Assuming equal access, in patients who are reasonable candidates to both BCMA CAR T-cell and TCE we recommend pursuing CAR T-cell therapy. This recommendation considers more robust data supporting activity of TCEs upon progression after CAR T-cell therapy and also the extended treatment-free interval post-CAR-T that is typically associated with more salvage options at the time of progression. However, as data on the use of combination therapies with TCE and sequential therapy with different antigen target evolves (for example talquetamab followed by cilta-cel), this will need to be revisited, especially in specific populations.
7. For patients with rapidly progressing disease and unlikely to transit through apheresis and bridging without disease-related morbidity, proceed with TCE due to faster access.
8. Both BCMA-targeted and GPRC5D-targeted immunotherapy are safe and active in patients with prior BCMA-targeted CAR T-cell therapy. Post BCMA-targeted CAR T-cell therapy, responses to BCMA-targeting therapies are likely less frequent and durable than in patients not previously treated with BCMA-targeted CAR T-cell therapy.
9. There are limited data on the feasibility and efficacy of BCMA-targeted therapy of a different modality upon progression on BCMA-targeted TCE at the approved dose intensities until progression. Outcomes after lower dose intensity or fixed duration of therapy are unknown. We recommend therapy with different mechanism of action or immunotherapy targeting a different antigen for patients progressing while receiving or shortly after receiving BCMA-targeting TCE.
